# Differential Expression of HPV16 L2 Gene in Cervical Cancers Harboring Episomal HPV16 Genomes: Influence of Synonymous and Non-Coding Region Variations

**DOI:** 10.1371/journal.pone.0065647

**Published:** 2013-06-06

**Authors:** Paramita Mandal, Bornali Bhattacharjee, Damayanti Das Ghosh, Nidhu Ranjan Mondal, Rahul Roy Chowdhury, Sudipta Roy, Sharmila Sengupta

**Affiliations:** 1 National Institute of Biomedical Genomics, Kalyani, West Bengal, India; 2 Human Genetics Unit, Indian Statistical Institute, Kolkata, India; 3 Department of Gynecology, Saroj Gupta Cancer Centre and Research Institute, Kolkata, India; 4 Department of Pathology, Kothari Medical Centre, Kolkata, India; IPO, Inst Port Oncology, Portugal

## Abstract

We tested the hypothesis that (i) synonymous variations within the coding regions, and (ii) variations within the non-coding regions of HPV, influence cervical cancer (CaCx) pathogenesis under the impact of intact HPV16 genomes. Whole genome sequence analysis of HPV16 isolates within 70 CaCx cases and 25 non-malignant samples revealed that synonymous variations were significantly higher within the E6 (p = 0.014), E5 (p = 0.001) and L2 (p = 0.0002) genes of HPV16 isolates within cases, compared to isolates within non-malignant samples. All of the 25 (100%) humanized codons identified within L2 ORF of the samples analyzed, were harbored by CaCx cases, while 8 out of 25 (32%) were harbored by HPV16 positive non-malignant samples (p = 3.87105E-07). L2 (mRNA and protein) expression was evident only among cases with episomal viral genomes and L2 mRNA expression correlated significantly with E2 gene copy numbers suggesting expression from all episomal genomes. Among such cases, Asian American (AA) isolates portrayed all of the humanized codons (100%; 4–6/sample) recorded within L2, which was significantly higher (p = 2.02E-7) compared to the European (E) isolates (22.8%; none or 1–2/sample). Additionally, majority of E variant isolates within cases (54/57; 94.7%) portrayed a variation (T4228C) within the short non-coding region (NCR2) between E5 and L2 genes, which portrays a weak promoter activity specific for L2 mRNA expression. This resulted in loss of 9 out of 14 miRNA binding sites (hsa-miR-548 family), despite the significant overexpression of miR548a-5p and miR548d-5p among such cases (28.64 and 36.25 folds, respectively), in comparison to HPV negative control samples. The findings exemplify the biological relevance of sequence variations in HPV16 genomes and highlight that episomal HPV16 in CaCx cases employ multiple mechanisms to sustain L2 expression, thereby justifying the potential role of L2 in such cancers, as opposed to those harboring viral integration.

## Introduction

The association of genital human papillomavirus (HPV) with cervical cancer (CaCx) is strong and independent of other risk factors, as evident from the consistent findings recorded from epidemiologic studies conducted in several countries [1]. Approximately 50% of CaCx cases are caused by HPV16 [2], [3]. In India also, HPV16 infection is the most predominant type associated with CaCx [4]–[6] and is also the most prevalent type identified in the general populations based on data available from some regions of India [5]–[9].

During the phase of transient infection, episomal form of HPV replicates along with the differentiating epithelial cells from basal membrane to the superficial zone, and viral particles therein are shed off along with the sloughed-off epithelial cells [10]. However, high grade cervical neoplasia appears to be characterized by deregulated viral gene expression and abortive life cycle of the virus [11]. Therefore, the transforming potential of HPVs are likely to be correlated with the potential of deregulating the expression of key viral proteins [12]–[14], as well as, with the ability to avoid immune attack by the host in order to persist within the host cervical epithelium [15].

Integration of viral genomes into the host genome, chiefly at fragile sites [16], [17], affects various cellular pathways of the host cell-cycle machinery. This leads to disruption of the viral E2 gene, most commonly in the region that encodes for hinge region of the HPV16 E2 protein. In absence of E2-driven repression, E6 and E7 are overexpressed, thereby driving infected cells towards transformation. On the contrary, our study [18] as well as a few others [19], have identified that a considerable proportion of individuals with CaCx harbor intact E2 gene [20]. This could be either purely intact (episomal) or concomitant, i.e., a mixture of intact (episomal) and disrupted (integrated) forms. Such observations, point towards the biological plausibility of cervical carcinogenesis under the impact of HPV16 intact E2 gene or intact viral genomes, as opposed to E2 disruption or integration.

In further exploration of novel paradigms of HPV16 related CaCx pathogenesis under the impact of episomal viral genomes with intact E2 genes, we undertook genome wide sequencing of such viral genomes within CaCx cases and non-malignant samples, initially excluding the E1 gene [21] and subsequently incorporating E1 in this study. Thus, we generated sequence data on the entire HPV16 genome. The European variant (E, 86.32%) was the most prevalent within our population both among controls as well as cases, followed by Asian–American variants (AA, 13.68%), which we recorded only among cases.

Nonsynonymous single nucleotide polymorphisms (SNP) are considered functional because they result in changes at the amino acid level that might functionally influence the proteins. Our previous analysis [21] was focussed on such variations within the most common E variant haplotype E-12, based on the SIFT database. This study revealed that rare deleterious variations within genes implicated in productive infection (L1, L2, E2 and E5), over the E-12 haplotype background of intact HPV16 isolates, might be of causal relevance for CaCx development. Synonymous variations on the other hand, could also influence viral gene expressions by modulating the codon usage patterns [22].

Earlier studies from our group have also provided an insight into the biological relevance of the non-coding regions of HPV16, such as the involvement of nucleotide variation within E2BSIV in the LCR [18], methylation of CpGs within E2BSI/II in the LCR [23] and repeat expansions within NCR-2 [21] in the pathogenesis of cervical cancers harboring intact HPV16 genomes. Our objective herein was to re-investigate the single nucleotide polymorphisms (SNPs) within the whole genome of HPV16, incorporating the E1 gene, among episomal HPV16 isolates within non-malignant samples and CaCx cases. Particularly, we emphasized on determining the association of synonymous variations within intact HPV16 genomes if any, with CaCx pathogenesis and identification of the genes that harbored such variations, in view of their biological relevance. We further explored the possibility that nucleotide variations within non-coding regions, specifically the untranslated regions of HPV16 genomes are biologically relevant as well, apart from those within coding regions.

## Materials and Methods

### Ethics Statement

All samples, malignant and non-malignant, were collected from the subjects with written informed consent approved by the institutional ethical committee for human experimentation of the Indian Statistical Institute, Kolkata, India.

### Samples and subjects

Details regarding subjects, samples, DNA isolation, HPV screening and determination of HPV16, E2 copy number and disruption status are described in details in our earlier studies [8], [18], [20], [21], [23], [24]. We analyzed DNA samples comprising of a panel of HPV16 positive malignant cases (n = 94) and HPV16 positive cytologically normal controls (n = 29), which we denoted here as HPV16 positive non-malignant samples. Of these, 70 malignant samples and 25 non-malignant samples have been included from our earlier report on HPV16 sequence data without the data on the E1 gene [21]. The malignant samples were characterized by median age of 50 years (range = 27–60 years) and the non-malignant samples by median age of 34 years (range = 27–80 years).

All the malignant samples (histopathologically confirmed invasive squamous cell carcinomas and clinically diagnosed as tumour stage III and above as per FIGO classification and majority were diagnosed as moderately differentiated squamous cell carcinoma pathologically) were derived from married subjects. The non-malignant samples were normal cervical scrapes confirmed by Pap smear test and derived from married and non-pregnant (or, 6 months post-partum) women with no previous history of cervical dysplasia/malignancy. A few of the samples from this group were histopathologically confirmed normal cervical biopsies derived from women undergoing hysterectomy for various reasons other than cancers such as uterine prolapse, fibroid, cyst etc. and without any prior history of cervical dysplasia/malignancy.

### Re-sequencing of HPV16 genome

The re-sequencing of HPV16 genomes was restricted to those samples (non-malignant and cases) harboring intact viral genomes based on (i) intact E2 gene as determined at the DNA level by PCR of the entire E2 gene [18] and (ii) Taqman assay for estimation of E2 and E6 gene copy numbers (episomal, when E2/E6 ratio≥1 and mixed or concomitant, when 0<E2/E6 ratio<1) [20].

Fifteen sets of overlapping primers were used for re-sequencing of HPV16 genome. Of these, the primer sequences and PCR conditions for eleven sets were described earlier from our laboratory [21]. In addition to these, four sets of overlapping primers were used spanning the entire region of the E1 gene. The details of primer sequences and PCR conditions for E1 gene are described in Table S1. Re-sequencing of the HPV16 intact genomes was done as described earlier [21] in an ABI Prism™3100 automated sequencer using dye terminator chemistry. The DNA sequences were analysed using the PolyPhred package (http://droog.mbt.washington.edu/PolyPhred.html) and HPV16R sequence was used as reference in the alignments [25]. Identification of rare variants and elimination of chances of sequencing errors were done as per the previous report from our group [21].

### Identification of biologically relevant synonymous variations within coding regions of HPV16 genome

The synonymous variations within the ORFs of HPV16 were determined from sequence data analysis. The frequency of usage of codons and amino-acids due to synonymous variations was identified based on the program “Graphical Codon Usage Analyzer (GCUA) available at http://gcua.schoedl.de/sequential_v2.html and finally humanized codons within the HPV16 ORFs were identified.

### Identification of biologically relevant variations within non-coding regions of HPV16 genome (short non coding region NCR2 between E5 and L2)

Nucleotide variations in the major non-coding region of HPV16, i.e. LCR were analyzed and reported earlier [18]. In the present communication, our focus was on the short non coding region, NCR2, between E5 and L2 regions of HPV16 in view of the possible involvement of this region in the regulation of L2 expression [26]. It has been identified recently that host miRNAs are able to impinge on viral life cycles, viral tropism, and the pathogenesis of viral diseases [27]. Therefore, using RegRNA (www.regrna.mbc.nctu.edu.tw/) software, we identified miRNA binding sites within the NCR2 of the HPV16 isolates and loss of such binding sites, if any, under the impact of single nucleotide variations. We further reconfirmed the loss of such binding, employing miRBase [28].

### RNA isolation and cDNA preparation

Total RNAs, from the cervical tissue samples were isolated, purified and treated with DNase I using the Qiagen RNeasy kit following the manufacturer's protocol. One microgram of total RNA from each sample was reverse transcribed using the primer (dT)17-P3, i.e., an oligo (dT)17-primer coupled to a linker sequence (5′GACTCGAGTCGACATCGA TTTTTTTTTTTTTTTTT 3′) [29] in a 20 µl reaction mix. In brief, each RNA sample was mixed with 400 ng of oligo-(dT)-P3 primer and incubated at 70°C for 10 minutes. The mix (10 µl) was quickly chilled on ice and then mixed with equal volume of a mixture of 2X reverse transcriptase buffer, 8 mM dNTPs (with dTT), 20 U RNase inhibitor and 50 U MultiScribe™ reverse transcriptase (High capacity cDNA Reverse Transcription kit, Applied Biosystems) and reverse transcribed at 42°C for 60 minutes followed by inactivation at 70°C for 10 minutes. Reverse transcription reaction, with mRNA and all reagents but no reverse transcriptase, was performed for the samples as negative controls.

### Quantitative PCR based analysis of L2 mRNA expression

The L2 mRNA expression was determined by quantitative PCR (qRT-PCR) on ABI 7900 HT PCR platform, following relative quantification with ACTB expression. For this assay, 100 ng of cDNA was used in a 10 µl reaction mixture with *Power* SYBR® Green PCR Master Mix (Applied Biosystems) and 25 ng of both forward (L2 (3) F: 5′ TAT GGA AGT ATG GGT GTATTT T 3′) and reverse primers (L2 (1) R: 5′ ATC TGG GGG AAT GGA AGG T 3′). ACTB expression was also quantified by real time PCR in a reaction volume of 10 µl including 100 ng of cDNA and 25 ng of forward (ACTB RTF: 5′ ATCCGCCGCCCGTCCACAC 3′) and reverse primers (ACTB RTR: 5′ TGCCGTGCTCGATGGGGTACT 3′). ACTB expression served as the internal control to ensure the integrity of the total RNA sample. Dissociation curve analysis was done, in order to rule out the occurrence of non-specific amplification and primer dimer formation. The PCR-controls were NTC (non-template control) as well as separate aliquots from Reverse Transcription reactions with (i) all reagents except mRNA, (ii) mRNA and all reagents but no Reverse Transcriptase, and (iii) HPV-negative cellular mRNA.

### Immunoblot analysis of L2 expression

Tissue samples (10 mg approximately) were homogenized in 100 µl ice cold protein lysis buffer (30 mM Tris HCl; pH = 7.5, 1 mM MgCl_2_, 1 mM EGTA, 0.67% β-mercaptoethanol, 0.5% CHAPs, 10% Glycerol and 0.5% Triton X100) containing protease inhibitor cocktail (Roche). After overnight incubation at 4°C with shaking, and subsequent centrifugation at 12,000 rpm at 4°C for 20 minutes, the supernatant was collected and estimated by Bradford assay (Biorad Hercules, CA) according to manufacturer's protocol. Thirty microgram of all protein samples were run on 12.5% SDS PAGE in duplicate and then transferred to PVDF membranes. After nonspecific blocking, the membrane was treated with 3∶5000 dilution of mouse L2 primary antibody (Santa Cruz Biotechnology, sc-65709; raised against amino acids 40–150 of HPV16 L2) overnight at 4°C. After washing, the membrane was again treated with anti-mouse secondary antibody (1∶5000 dilution, goat anti-mouse IgG-HRP, Santa Cruz Biotechnology, sc-2005) at 37°C for 2 hours and 30 minutes. The L2 protein expression was detected by chemiluminiscence based assay, after washing the membrane. Expression of ACTB protein was determined as internal control. Mouse monoclonal ACTB primary antibody (2∶5000 dilution, Abcam, ab6276) and anti-mouse secondary antibody (1∶5000 dilution, goat anti-mouse IgG-HRP, Santa Cruz Biotechnology, sc-2005) were used for ACTB protein expression analyses. Densitometric analysis of each band of L2 and ACTB were performed using IMAGE J software (http://rsb.info.nih.gov/ij/docs/index.html). L2 protein expression was represented in terms of relative density of each band of L2 normalized with the corresponding ACTB protein band (area of L2 protein band/area of ACTB protein band).

### Relative quantification of mature miRNAs by TaqMan miRNA real-time PCR

TaqMan MiRNA Assays for miR-548a-5p and miR-548d-5p were undertaken, employing cDNA prepared from total RNA samples, using specific miRNA primers from the TaqMan MiRNA Assays and reagents from TaqMan® MiRNA Reverse Transcription Kit (ABI; Cat#4366596). The 15 µl reverse transcription reactions consisted of 10 ng of total RNA, 5 U MultiScribe Reverse Transcriptase, 0.5 mM of each dNTP, 1× reverse transcription buffer, 4 U RNase inhibitor, and nuclease-free water. This was performed at 16°C for 30 min and at 42°C for 30 min, terminated at 85°C for 5 min. For real-time PCR of TaqMan MiRNA Assays, we used 0.5 µl 20×TaqMan MiRNA Assay Primer, 1.33 µl undiluted cDNA, 5 µl 2×TaqMan Universal PCR Master Mix and 3.17 µl nuclease-free water. The real time PCR program included initial denaturation at 95°C for 10 minutes, followed by 40 cycles of denaturation at 95°C for 15 seconds and annealing at 60°C for 1 minute. The PCR-controls were NTC (non-template control). Each assay was performed at least twice, with three replicates per sample in each assay, on MicroAmp optical 96-well plates using a 7900 HT PCR System (ABI). Relative expression of the miRNAs were calculated using RNU6b (TaqMan miRNA control assay) as the endogenous control, and calibrated to the control samples.

### Statistical analyses

The association of the various nucleotide changes within the viral genome, with CaCx pathogenesis, were determined using chi-square test as appropriate. For this we compared between the cases and non-malignant group after adjusting for size of the respective ORFs. False discovery rates of 0.05 were obtained to correct for multiple testing using the Benjamini and Hochberg's method [30].The difference in the percentage of humanized codons and SNPs in NCR2 between CaCx cases and non-malignant samples, and between AA and E variants was also determined by chi-square test. L2 mRNA expression and densitometry based analysis of L2 protein expression data was expressed as mean ± standard deviation. Kolmogorov-Smirnov test was performed to identify whether the test variables like expression of L2 mRNA and protein, followed normal distribution. Two sample t-test was used to identify association of disease phenotype with variables that followed normal distribution. A p value less than 0.05 was considered statistically significant. Linear regression analysis was performed to determine the association of E2 copy numbers with L2 mRNA expression. Box plots were constructed to observe the difference in distribution of miRNAs expressions among different categories of cervical samples. Kolmogorov–Smirnov test identified miRNAs expression as a variable not following normal distribution. Therefore, non-parametric test (Mann–Whitney U test) was performed to study association of miRNAs expression with the disease phenotype. All statistical analyses were done using software packages SPSS (version 16.0 for windows) and R (www.r-project.org).

## Results

### Nucleotide variations within E1 ORF: type and frequency

The nucleotide variations within the ORFs of HPV16 genome, except for E1, have been reported earlier from our laboratory [21]. Single nucleotide variations were recorded at 20 positions within E1 ORF (Table 1). Of the single nucleotide variations, 19 were bi-allelic changes barring one, which was tri-allelic. The frequency of variations ranged between 0.01 and 0.45 and on the basis of minor allele frequencies (MAFs) were classified as polymorphisms (MAF≥0.05) and low frequency variations (MAF<0.05). Of the 20 variations within the ORF, there were 9 (45%) non-synonymous variations and 11 (55%) synonymous variations distributed across the E1 gene.

**Table 1:** Nucleotide sequence variations and amino acid changes within the E1 ORF of intact HPV16 isolates among the samples (non-malignant samples and CaCx cases) analysed.

Single nucleotide Variations	MAF	Codon change	Amino acid change	Position
C874T	0.021	CCT-TCT	P-S	4
T921C	0.137	TTT-TTC	F-F	19
T1221C	0.063	AGT-AGC	S-S	119
G1293T	0.379	CAG-CAT	Q-H	143
T1297A	0.01	TTA-ATA	L-I	145
G1363A	0.074	GGT-AGT	G-S	167
T1421C	0.211	ATA-ACA	I-T	186
T1446C	0.032	ATT-ATC	I-I	194
A1842G	0.137	ATA-ATG	I-M	326
T1920G/C	0.021/0.042	GAT-GAG/GAC	D-E/D	352
G1941A	0.021	CAG-CAA	Q-Q	359
T2301C	0.01	GCT-GCC	A-A	479
G2337A	0.01	ATG-ATA	M-I	491
T2343C	0.053	TTT-TTC	F-F	493
C2344T	0.137	CTG-TTG	L-L	494
T2470C	0.032	TTA-CTA	L-L	536
T2478C	0.01	AAT-AAC	N-N	538
G2650A	0.053	GAA-AAA	E-K	596
T2778C	0.042	TTT-TTC	F-F	638

### Non-synonymous amino acid changes across all ORFs of HPV16 genome

Earlier, we recorded 110 non-synonymous variations distributed across the ORFs of HPV16, excepting E1 [21]. Such variations remained unaltered, even after increasing the sample size to 70 cases and 25 non-malignant samples. Thus, our whole genome sequence analysis of HPV16 intact viral genomes revealed a total of 119 non-synonymous variations. The percentage of such variations within E1 was not significantly different between cases (0.08%) and non-malignant samples (0.11%). Multiple testing corrections were done, after including the non-synonymous variations within E1 ORF together with those of the other ORFs. Such analysis re-confirmed that percentage of non-synonymous variations in L2 ORF was significantly higher in cases, compared to HPV16 positive non-malignant group (Table 2).

**Table 2:** Distribution of non-synonymous variations between non-malignant samples and CaCx cases across the coding regions of the HPV16 intact isolates.

ORF	Size(bp)	Variations within CaCx cases (n = 70)		Variations within non-malignant samples (n = 25)		p-value	FDR of 0.05
		Non synonymous	%	Non synonymous	%		
E4	287	100	0.5	27	0.38	0.195	0.05
E1	1949	120	0.08	55	0.11	0.124	0.0429
E6	455	68	0.21	15	0.13	0.088	0.0357
L1	1517	60	0.06	12	0.03	0.063	0.0286
E2	1097	150	0.2	36	0.13	0.031	0.0214
E5	251	138	0.79	32	0.51	0.026	0.0143
**L2**	**1421**	**149**	**0.15**	**20**	**0.06**	**2.78E-05**	**0.0071**

The percentage of non synonymous variations was estimated on the basis of total number of non synonymous variations out of total number of nucleotides (normalized with the size of the ORFs) in cases or non-malignant samples within the respective ORFs; *e.g.* For E4: % within cases = [100/(287×70)]×100 and % within non-malignant samples = [27/(287×25)]×100.

***Bold emphasis indicates statistically significant p-values.***

### Synonymous amino acid changes and humanized codons across the various ORFs of HPV16 genome

A total of 124 synonymous variations were recorded distributed across the coding regions of HPV16 genomes of intact isolates. The percentage of synonymous variations were significantly higher in cases compared to non-malignant samples for E6 (cases = 0.104%, non-malignant samples = 0.026%, p = 0.014), E5 (cases = 0.296%, non-malignant samples = 0.064%, p = 0.001) and L2 (cases = 0.22%, non-malignant samples = 0.121%, p = 0.0002) ORFs (Table 3). Further analysis was performed, by using the GCUA tool (http://gcua.schoedl.de/sequential_v2.html), to identify the humanized codons within E5, E6 and L2 ORFs under the impact of synonymous variations.

**Table 3:** Distribution of synonymous variations between non-malignant samples and CaCx cases across the coding regions of the HPV16 intact isolates.

ORF	Size(bp)	Variations within CaCx cases (n = 70)		Variations within non-malignant samples (n = 25)		p-value	FDR of 0.05
		Synonymous	%	Synonymous	%		
E7	297	48	0.23	10	0.13	0.116	0.05
E2	1097	75	0.098	17	0.062	0.088	0.04375
E4	287	58	0.289	12	0.167	0.081	0.0375
E1	1949	47	0.034	8	0.016	0.047	0.03125
L1	1517	153	0.144	30	0.08	0.044	0.025
**E6**	**455**	**33**	**0.104**	**3**	**0.026**	**0.014**	**0.01875**
**E5**	**251**	**52**	**0.296**	**4**	**0.064**	**0.001**	**0.0125**
**L2**	**1421**	**222**	**0.22**	**43**	**0.121**	**0.0002**	**0.00625**

The percentage of synonymous variations was estimated on the basis of total number of synonymous variations out of total number of nucleotides (normalized with the size of the ORFs) in cases or non-malignant samples within the respective ORFs; e.g. For E4: % within cases = [58/(287×70)]×100 and % within non-malignant samples = [12/(287×25)]×100.

***Bold emphasis indicates statistically significant p-values.***

There were 25 humanized codons in L2 and 2 such codons in both E5 and E6 (Table S2). It was observed that all of the 25 (100%) humanized codons identified within L2 ORF of the samples analyzed, were harbored by CaCx cases, while 8 out of 25 (32%) were harbored by HPV16 positive non-malignant samples. Thus the frequency of humanized codons in L2 ORF was significantly higher (p = 3.87105E-07) in CaCx cases, compared to HPV16 positive non-malignant samples. No significant differences were found in the frequencies of humanized codons in E5 and E6 ORFs, between CaCx cases and HPV16 positive non-malignant samples (Table 4).

**Table 4:** Distribution of percentage of humanized codons between non-malignant samples and CaCx cases across the E5, E6 and L2 ORFs.

ORFs	No. of codons	Humanized codons		Proportion of humanized codons within cases	Proportion of humanized codons within non-malignant samples	p-value	FDR of 0.05
		*No*	*%*				
E5	84	2	2.381	2/2(100%)	2/2(100%)	1	0.05
E6	152	2	1.316	2/2(100%)	1/2(50%)	0.248	0.033
**L2**	**474**	**25**	**5.27**	**25/25 (100%)**	**8/25(32%)**	**3.87E-07**	**0.017**

***Bold emphasis indicates statistically significant p-values.***

We further classified the HPV16 intact isolates into E and AA variants following a classification scheme as reported earlier from our laboratory [21]. After inclusion of additional samples in this study we failed to record AA variants among the non-malignant samples, while among the E2 intact CaCx cases, the proportion of AA and E variants was 18.6% (13/70) and 81.4% (57/70), respectively. We therefore made an attempt to compare AA and E variants in terms of humanized codons in L2 ORF among CaCx cases only. Our analysis revealed that all AA variants (13/13,100%) harbored humanized codons in the L2 region whereas only few E variants (13/57, 22.8%) harbored such codons and this difference was statistically significant (p = 2.02E-7). The number of humanized codons was also distinctly different between the two variants. Characteristically, each AA variant harbored 4–6 humanized codons in contrast to each E variant that harbored none or a maximum of 2 humanized codons.

### Differential expression of L2 mRNA among CaCx cases harboring episomal (pure or concomitant) and integrated HPV16 genomes

**Figure 1:**
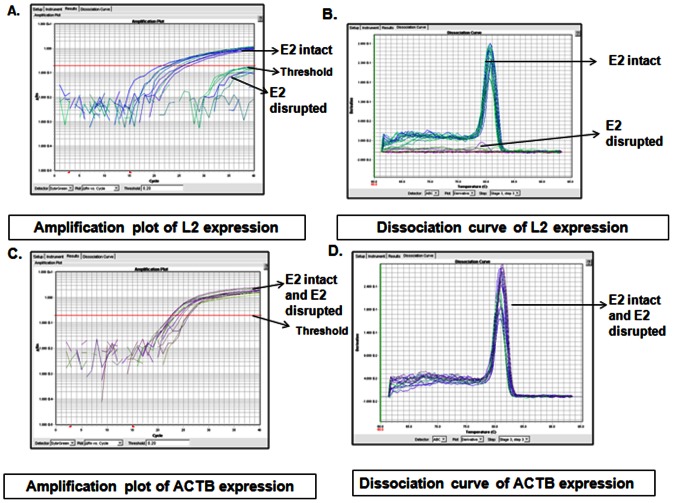
Relative quantification of L2 mRNA expression. (**A**) Amplification plot based on quantitative real time PCR of HPV16 L2 expression. L2 is transcribed in E2 intact/episomal (episomal or concomitant) but in E2 disrupted/integrated cases. (**B**) Dissociation curve depicting the first-derivative melting curve for the reaction characterizing the expression of L2 (Tm of 80.5°C). (**C**) Amplification plot based on quantitative real time PCR of ACTB expression. ACTB is expressed by both episomal and integrated HPV16 positive cases. (**D**) Dissociation curve depicting the first-derivative melting curve for the reaction characterizing the expression of ACTB (Tm of 81.0°C).

**Figure 2:**
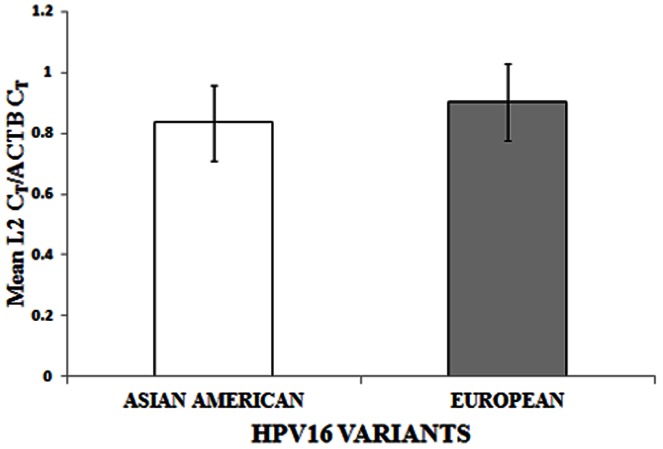
L2 mRNA expression among Asian American (n = 7) and European (n = 16) variants within CaCx cases with episomal HPV16 normalized to ACTB expression. Relative L2 mRNA expression is represented by mean L2 C_T_/ACTB C_T_.

**Figure 3:**
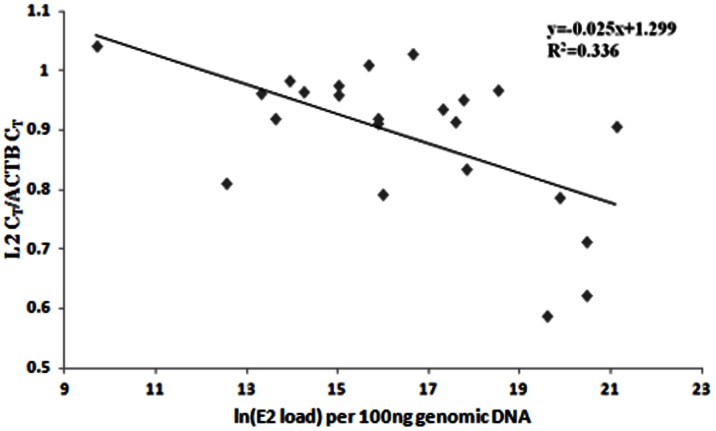
Linear regression analysis of the correlation between L2 mRNA expression normalized with ACTB mRNA expression (L2 C_T_/ACTB C_T_) and E2 load (E2 copy number) per 100 ng genomic DNA (natural log values) in CaCx cases with E2 intact/episomal (episomal and concomitant) viral genomes(p = 0.004).

In our earlier study, we confirmed the intactness of the E2 gene by analysing the presence of the viral transcript (E7-E1∧E4) that produces the repressor E2, by APOT (amplification of papillomavirus oncogenic transcript)-coupled-quantitative-RT-PCR of E7 and E4 (nested to the E2 gene) genes [31]. Based on such analysis, samples were classified as pure episomal or concomitant (episomal and integrated) with intact E2 genes, and integrated with disrupted E2 genes. The study [31] also revealed that these two types of cancers differed in the expression of E7 and E2 mRNAs. We therefore determined L2 mRNA expression, by quantitative real time PCR on 23 episomal/concomitant HPV16 positive CaCx cases, and compared the data with that of 11 integrated CaCx cases. No L2 expression was recorded among the integrated cases, as opposed to distinct L2 mRNA expression in episomal/concomitant CaCx cases (Figure 1), which was quite similar to that recorded in case of E2 expression in our earlier study [31]. All of the samples analysed, portrayed the expression of ACTB mRNA transcripts as internal control. Further analysis failed to reveal significant (p = 0.224, t-test) differences in L2 mRNA expression between AA [mean (L2 C_T_/ACTB C_T_) ± sd = 0.834±0.127] and E [mean (L2 C_T_/ACTB C_T_) ± sd = 0.904±0.128] variants (Figure 2). The ratio, L2 C_T_/ACTB C_T_, was also found to be significantly correlated with the E2 copy numbers (p = 0.004; R^2^ = 0.336) within the episomal CaCx cases (Figure 3), justifying the expression of L2 from episomal viral genomes.

### Differential expression of L2 protein among CaCx cases harboring episomal (pure or concomitant) and integrated HPV16 genomes

**Figure 4:**
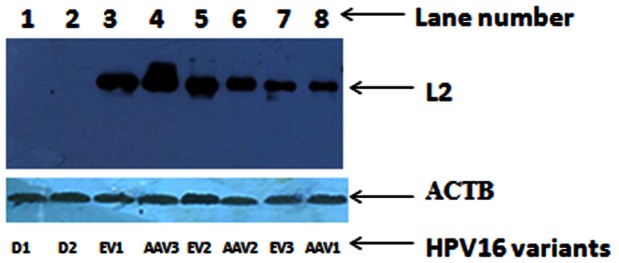
Representative Immunoblot analysis of L2 and ACTB protein expression. Upper panel depicts L2 expression. Lanes 1 and 2: HPV16 positive E2 disrupted/integrated CaCx case samples (D1 and D2); Lanes 3, 5, and 7: HPV16 positive E2 intact/episomal (episomal or concomitant) European variants (EV1, EV2, EV3, respectively); Lanes 4, 6, and 8: HPV16 positive E2 intact/episomal (episomal or concomitant) Asian American variants (AAV3, AAV2, AAV1, respectively). Lower panel depicts ACTB expression among all the samples analysed. Sample details are illustrated in Table 5.

**Figure 5:**
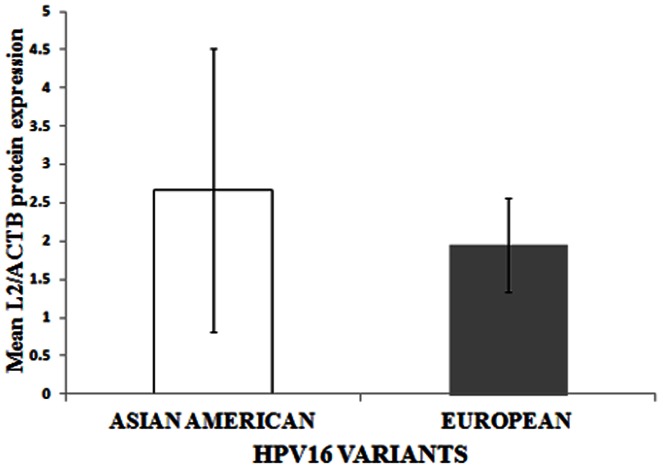
Densitometric analysis of L2 protein expression (normalized with ACTB expression), among HPV16 positive E2 intact/episomal (episomal or concomitant) CaCx cases harboring Asian American and European variants.

We determined L2 protein expression by immunoblot analysis, on a subset of 12 CaCx cases (integrated or E2 disrupted CaCx cases = 4, Asian American episomal or E2 intact CaCx cases = 3 and European episomal or E2 intact CaCx cases = 5) from the set that was used for L2 mRNA expression analysis. L2 expression was recorded among the episomal CaCx cases, AA and E variant isolates, while such expression could not be identified among the integrated CaCx cases (Figure 4). All the CaCx samples, irrespective of episomal or integrated, portrayed the expression of ACTB protein (endogenous control). The status of humanized codons within the AA and E variant isolates of samples revealing L2 protein expression is depicted in Table 5. L2 protein expression was quantified by densitometric analysis of immunoblot results by IMAGE J software (http://rsb.info.nih.gov/ij/docs/index.html) and no significant difference (p = 0.562, t-test) was recorded between AA [mean (area of L2 protein band/area of ACTB protein band) ±sd = 2.66±1.85] and E variants [mean (area of L2 protein band/area of ACTB protein band) ± sd = 1.95±0.61] as portrayed in Figure 5.

**Table 5:** Number of humanized codons within the AA and E variant isolates of CaCx samples analyzed for L2 protein expression.

Samples	HPV16 variants	Number of humanized codons in L2 gene
AAV1	Asian American	4
AAV2	Asian American	5
AAV3	Asian American	6
EV1	European	1
EV2	European	None
EV3	European	None

### miRNA binding sites in the short non coding region (NCR2) and loss of such binding sites due to presence of SNPs in the NCR2 of CaCx cases

**Figure 6:**
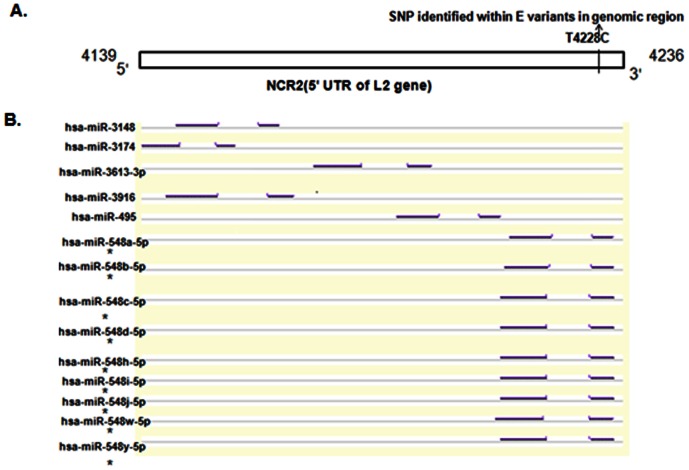
miRNA binding sites and variant nucleotide position within NCR2 of E2 intact/episomal (episomal or concomitant) HPV16 European (E) variant isolate within CaCx cases. (**A**) Depicts the NCR2 (nucleotide positions 4139–4236) located within 5′ UTR of L2 gene, with a single nucleotide polymorphism (SNP) at position 4228 (T to C). (**B**) RegRNA software based identification of fourteen miRNA binding sites within NCR2 with loss of binding sites corresponding to nine miRNAs (_*_) of the hsa-miR-548family due to the SNP (T4228C).

A short non-coding region (NCR2) commonly exists between the E5 and L2 open reading frames of HPVs. NCR2 is characterized by a weak promoter activity that is tightly regulated by keratinocyte differentiation and used only for transcripts encoding the minor capsid protein L2 of HPV16 [26]. Another study reported 13 transcripts of HPV16 in cervical epithelial cell line W12 (harboring episomal HPV16 genomes), of which, 6 transcripts were found to encompass the NCR2 and L2 [32]. In view of the fact that the CaCx cases harboring episomal HPV16 genomes also expressed the L2 gene, we focussed on deciphering the factors that could be associated with L2 expression in such CaCx cases. By using RegRNA (www.regrna.mbc.nctu.edu.tw/) software, we identified binding sites in the NCR2 (nt 4139–4234) of HPV16 intact isolates, corresponding to 14 human miRNAs (hsa-miR-3148,hsa-miR-3174,hsa-miR-3613-3p,hsa-miR-3916,hsa-miR-495,hsa-miR-548a-5p, hsa-miR-548b-5p, hsa-miR-548c-5p, hsa-miR-548d-5p, hsa-miR-548h-5p, hsa-miR-548i-5p, hsa-miR-548j-5p, hsa-miR-548w-5p, hsa-miR-548y-5p) (Figure 6). Such miRNA binding sites were selected on the basis of minimum free energy (MFE≤7) and hybridization score (≥140) (Table S3) as per standards normally used for formation of miRNA:mRNA hybrid. Our resequenced data revealed the occurrence of a SNP (T4228C) (Figure S1) in the NCR2 of E variant intact isolates only, which could lead to loss of 9 miRNA binding sites in the corresponding transcripts (hsa-miR-548a-5p, hsa-miR-548b-5p, hsa-miR-548c-5p, hsa-miR-548d-5p, hsa-miR-548h-5p, hsa-miR-548i-5p, hsa-miR-548j-5p, hsa-miR-548w-5p, hsa-miR-548y-5p) (Figure 6), all of which belonged to the hsa-miR-548 family of miRNAs. Interestingly, proportion of E2 intact CaCx cases (54/70, 77%) harboring SNPs in the miRNA binding sites within the NCR2 was significantly higher (p = 0.007) compared to that of non-malignant samples (12/25, 48%). Within E2 intact CaCx cases, it was also observed that none of AA the variants (0/13, 0%) harbored a SNP in the miRNA binding sites in the NCR2. Thus, no loss of miRNA binding sites in the NCR2 was observed in AA variants.

Previous study from our laboratory [21] revealed the occurrence of repeat variations within the NCR2. In view of the fact that such variations as well as the SNP (T4228C) in NCR2, could have an impact on L2 mRNA as well, we employed RNA fold and SNP fold algorithms to predict minimum free energy for determination of the stability of the ensemble of late transcripts that incorporated NCR2 and L2 sequences in such HPV16 positive (AA and E variant isolates) CaCx cases harboring episomal viral genomes. The minimum free energy was almost similar among the E and AA variant isolates with or without repeat variations or SNPs, which confirmed that the ensemble of L2 mRNA encoding transcripts structure and stability was not affected by such variations (Table S4 and Figure S2).

### Differential expression of miR-548a-5p and miR-548d-5p among CaCx cases harboring episomal (pure or concomitant) and integrated HPV16 genomes

**Figure 7:**
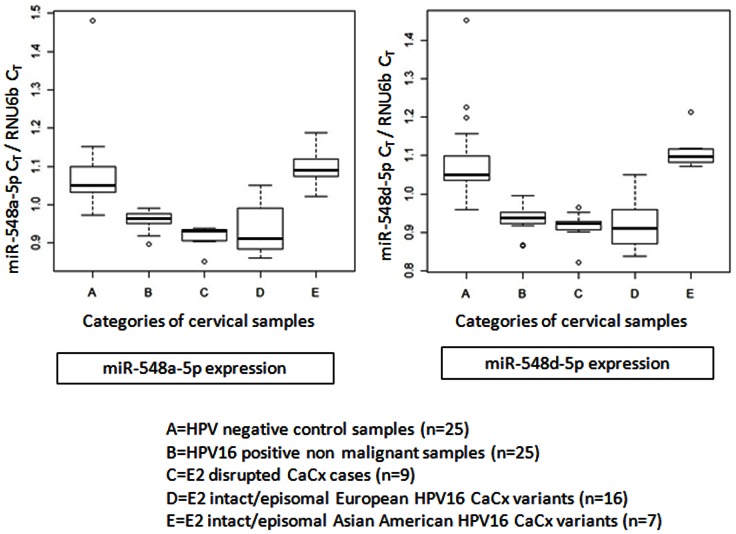
Box plots representing distribution of miR-548a-5p and miR-548d-5p expression (normalized with RNU6b expression as endogenous miRNA control) among different categories of cervical samples.

Of the 9 miRNAs, for which we recorded loss of binding sites within the NCR2 of episomal HPV16 E variant isolates, we randomly selected two miRNAs (miR-548a-5p and miR-548d-5p) to test the hypothesis that they are expressed but deemed non-functional. Our analysis revealed that miR-548a-5p and miR-548d-5p were significantly upregulated (p<0.001, Mann Whitney U test) in HPV positive non-malignant samples (8.51 and 18 folds respectively), E2 disrupted CaCx cases with integrated viral genomes (22.01 and 22.32 folds respectively) and CaCx cases with episomal E variant of HPV16 (28.64 and 36.25 respectively). This was recorded in comparison to HPV negative control samples. No such significant difference was observed in CaCx cases with episomal AA variant isolates, in comparison to HPV negative control samples. Such findings are depicted in Figure 7.

## Discussion

We undertook the present study in pursuit of exploring alternative mechanisms of CaCx pathogenesis in the presence of episomal HPV16 harboring intact E2 genes [23]. In continuation of our earlier study [21] on the impact of non-synonymous variations on disease risk, in this study we undertook an in depth analysis of synonymous variations within the coding regions and variations within the non-coding and/or UTRs of intact HPV16 isolates within CaCx cases as compared to those within HPV16 positive non-malignant samples. We further focussed on the two major viral lineages of HPV16, i.e. E and AA variants, in order to decipher whether such viral isolates in CaCx cases follow similar or different mechanisms of disease pathogenesis, with respect to such variations. It is established that both types exist in the episomal form among CaCx cases [21].

On completion of the whole genome sequencing of HPV16 on an enhanced sample set upon inclusion of E1, we reanalysed the data on non-synonymous variations across all the ORFs. None of the variations of the E1 gene appeared to be significantly associated with CaCx pathogenesis. This appears to be in contrast to a recent study on Chinese population. This study employed a whole genome sequencing based approach, which identified a positively selected site 491 in the E1 protein located within the E2 binding domain that was capable of binding to DNA polymerase alpha-Primase p68 Subunit [33]. Other studies, focusing on E1 sequence variation analysis on Croatian [34] and Slovakian populations [35], identified a 63-bp in-frame insertion in the E1gene. Such studies predicted that this change might have reduced pathogenicity, compared to the E1 wild type strains. Further to our earlier study [21], based on this whole genome association analysis, we reconfirmed that non-synonymous variations were significantly higher within the L2 gene of CaCx cases with intact E2, as opposed to the non-malignant samples. The L2 gene could therefore play a significant role in mediating CaCx pathogenesis.

Synonymous variations within coding regions of viral genomes are likely to influence viral protein expressions through codon usage bias, thereby exerting an effect on disease pathogenesis. Codon usage bias refers to differences among organisms in the frequency of occurrence of codons in protein coding DNA sequences. This bias in codon preference has been reported in most genomes that have been studied so far [36]. In some organisms, highly expressed genes revealed a strong codon preference consistent with the concentrations of corresponding tRNAs, whereas genes expressed at lower levels portrayed a more uniform pattern of codon use [37]. Viruses rely on their host's cell machinery to transcribe and translate their genes. Hence the abundance of tRNAs in the host, dictates the efficiency with which viral RNA is translated into protein [22]. Consequently, it is hypothesized that host tRNA abundance leads to codon bias in viruses [38]–[40], and that host shift is constrained by codon usage patterns. Thus, viral virulence could be attributable partially to this codon bias, and most virulent strains could be those that match the codon usage patterns of the respective host genomes.

Our study further revealed that the percentage of humanized codons as a consequence of synonymous variations, were also significantly higher in CaCx cases compared to non malignant samples, only for the L2 gene. This was despite the fact that there was overrepresentation of synonymous variations in E5 and E6 genes as well, along with L2. This observation further highlighted the potential involvement of L2 gene in CaCx pathogenesis, when the viral genome appeared in the episomal form within the cervical tissues. Further support of this hypothesis was derived from the observation of L2 expression, both at the mRNA and protein levels, in E2 intact CaCx cases with episomal HPV16 genomes. This was irrespective of HPV16 lineage, and in contrast to absence of such expression in E2 disrupted CaCx cases with integrated viral genomes. Our next attempt was to explore, whether the underlying mechanisms likely to be associated with L2 expression in the CaCx cases with episomal HPV16 genomes, were similar or different for AA and E variants of HPV16.

Characteristically, the AA variants among CaCx cases with episomal HPV16 portrayed all (100%) of the humanized codons recorded within L2. This was in contrast to E variants, which portrayed only 69.51% of such codons. Besides, the number of humanized codons were higher in AA variants (4–6 per sample) compared to E variants (none or 1–2 per sample). There was a positive correlation (R^2^ = 0.98) between the number of humanized codons with L2 protein expression in AA variant cases only (data not shown). Thus, while synonymous nucleotide variations leading to humanized codons in L2 justify the L2 expression in AA variant cases only, there could be alternative mechanisms associated with L2 expression in episomal E variant CaCx cases. Our next attempt was therefore, to explore whether variations within non-coding regions of HPV16 intact isolates could influence L2 expression among such cases.

The role of LCR in the biology of HPV infections is well characterized. The NCR2 in HPV16 genome, characterized by a weak promoter activity [26], is known to be tightly regulated by keratinocyte differentiation. This is used only for transcripts encoding the minor capsid protein L2 [26], as observed in several previous studies [41]–[44]. There are reports suggesting, that UTRs are readily transcribed and they may play a central role in posttranscriptional regulation by being integral to the mature mRNA [45]. Strikingly, we identified the existence of fourteen human miRNA binding sites (Figure 6) in this NCR2 region of HPV16 intact isolates, irrespective of case or non-malignant samples. Thus, loss of binding sites corresponding to nine such miRNAs, due to a SNP (T4228C) in the NCR2 region of cases with episomal E variant isolates (Figure 6), could potentially serve as a novel mechanism facilitating the expression of L2 in such variant cases. This could be complementary to the few humanized codons recorded among the E variants, in contrary to the major role of humanized codons in case of L2 expression among cases with episomal AA variant isolates.

miRNAs, through recognition of sequence-complementary target elements, can either translationally suppress or catalytically degrade both cellular and viral RNAs [46], [47]. The NCR2 in HPV16 genome, by virtue of being located at the 5′ region of L2 gene, is supposed to be a 5′ untranslated region (UTR) of the L2 gene. miRNAs are mostly known to bind to the 3′ untranslated regions (UTRs) of their target mRNAs and interfere with translation. A recent study demonstrated that mRNAs are repressed as efficiently by miRNA binding to sites in the 5′UTRs, as in case of the 3′UTRs [48]. Our study thus clearly illustrates the possibility of host cellular miRNAs targeting HPV16 mRNAs at the 5′UTR of L2 gene, which, the episomal E variant isolates in CaCx cases overcome through nucleotide variations in the NCR2 region. We therefore tested the possibility of altered expression of miR-548 family (in HPV16 related CaCx cases) by determining the expression of miR-548a-5p and miR-548d-5p through quantitative PCR based assay.

We recorded a progressive upregulation of such miRNAs from HPV16 positive non-malignant samples to HPV16 positive CaCx cases (with integrated HPV16 and episomal E variant isolates), barring the HPV16 positive cases with AA variant isolates (Figure 7). This appears to be the first report establishing a novel role of miR-548 family in HPV16 related CaCx pathogenesis by targeting the viral genomes at the NCR2 and restricting the viral L2 gene expression, as observed in CaCx cases with integrated viral genomes. However, lack of binding of such miRNAs to the NCR2 of cases with episomal E variant isolates, despite their overexpression, could also be biologically relevant for cervical carcinogenesis. We therefore speculate that in such cases, where L2 protein potentially plays an oncogenic role, overexpression of miRNA-548 family could play a complementary role in supporting the oncogenicity of the episomal E variant isolates. This is based on a recent *in vitro* study demonstrating that miRNA-548 down-regulates host antiviral response via direct targeting of IFN-λ1 [49].

Interestingly, the CaCx cases with episomal AA variant isolates neither portray such variations within the NCR2, nor reveal overexpression of miR-548a-5p and miR-548d-5p, further strengthening the role of L2 expression in maintaining oncogenic status among such cases under the impact of humanized codons. Humanized codons in HPV are known to be established as a means by which, the virus overcomes the translational blockage and weak expression of both HPV capsid genes and oncogenes in undifferentiated epithelial cells [50]. This could, in turn, support HPV persistence and oncogenic status of the cervical epithelium. Furthermore, it has also been observed that the L2 protein is involved in the induction of immune escape of HPV16 through the manipulation of Langerhans cells [51].Taken together, our observations appear to be in line with the hypothesis that the ability to avoid immune attack is also linked to the transforming potential of papillomaviruses [52], applicable for both the E and AA variant episomal isolates in CaCx cases.

The 5′UTRs of viral mRNAs have also been demonstrated to regulate translation efficiency, by forming secondary structures and interacting with internal ribosome entry sites, thereby positively modulating viral gene expression [53]. We excluded such a possibility for L2 expression among CaCx cases harboring episomal HPV16 genomes in the light of the SNP (T4228C) and repeat variations [21] within the NCR2 region of E variant isolates and repeat variations only in case of AA variant isolates, based on an *in silico* approach (Table S4). Likewise, we also examined other factors that might influence L2 gene expression in CaCx cases harboring episomal HPV 16.

During the productive life cycle of HPV infections in the cervical epithelium, L1 and L2 protein expression is confined to the upper epithelial layers and is regulated post-transcriptionally in response to epithelial differentiation. A 79 nt RNA regulatory element (7128–7206 nt), the late regulatory element (LRE) involved in this regulation, is located at the 3′ end of the L1 gene and extends into the late 3′ UTR. This element represses late gene expression in undifferentiated epithelial cells and activates such expression in the uppermost terminally differentiated cells of the epithelium [54]. We recorded for the first time, expression of L2 protein in CaCx cases harboring episomal HPV16 genomes. This prompted us to reinvestigate the sequence of this non-coding region, LRE, subsequent to our previous report [21] that demonstrated a nucleotide variation (G7193T) in this region (Figure S3). Through whole genome sequence analysis of HPV16 in this study, we reconfirmed the presence of this SNP and recorded that about 88.6% (62/70) of the E2 intact cases harbored this variation, as opposed to none of the E2 disrupted cases (data not shown) harboring this variation. Thus loss of LRE mediated repression, under the impact of this sequence variation, could also potentially influence L2 expression in CaCx cases harboring episomal viral genomes, in addition to loss of miRNA binding sites in the 5′UTR of L2 gene of E variant isolates.

Besides identifying the biological relevance of sequence variations, both in the coding and non-coding regions of the HPV16 episomal viral genomes within CaCx cases, our study also highlighted the possibility of an interactive role of L2 and E2 proteins in such cases. We recorded a significant positive correlation between E2 gene copy numbers and L2 mRNA expression in CaCx cases harboring episomal viral genomes, confirming the expression of L2 from all such episomal viral genomes. A recent study from our laboratory [31] identified the expression of E2 from episomal viral genomes, which failed to induce repression of E7 as a result of methylation within CpGs at the E2 binding sites I and II. However, E2 remained functional in terms of replication and segregation as evident from the occurrence of high viral load in such cases harboring episomal HPV16 genomes, compared to those with integrated viral genomes. A novel function of E2 has recently been identified [55], revealing that it contributes to induction of HPV16 late gene expression by causing a read-through at the early polyadenylation signal (pAE) into the late region of the HPV genome. Inhibition of pAE by E2 protein involving the N-terminal and hinge regions has also been confirmed in vitro [55]. Thus, CaCx cases with episomal as opposed to integrated HPV16 probably sustain expression of L2 at the cost of E2 expression, by overcoming early polyadenylation as well.

The expression of L2 protein in CaCx cases harboring intact or episomal HPV16 genomes, thus appears to be indispensible, albeit its unknown role in supporting the oncogenic status of such cases. It is established from earlier studies that in productive phase of viral life cycle, subsequent to viral entry and shedding of the viral coat, the transfer of viral DNA to the host cellular nucleus is mediated by the minor capsid protein L2 [56], [57]. L2 also plays a major role in encapsidation of the viral genome into the capsid during virion formations within the nucleus. During such processes, L2 of HPV16 interacts with several cellular host proteins [58], recruiting one of them to the nucleus [59], and is complexed with cellular proteins in specific nuclear domains. These findings suggest the likelihood of a modulatory influence of L2 on host-cell functions involving discrete nuclear domains, and alteration of the subcellular distribution of cellular proteins. This calls for identification of the interacting cellular proteins of L2, which might influence the viral life cycle by facilitating viral persistence and expression of viral oncogenes, in order to maintain the malignant phenotype in such CaCx cases harboring episomal viral genomes.

## Conclusion

Our study exemplifies the biological relevance of synonymous sequence variations as well as those variations that are located within non-coding regions of HPV16 genomes, in CaCx pathogenesis. The L2 gene appears to be the hot-pot of such variations, culminating into multiple routes employed by episomal HPV16 in CaCx cases to sustain L2 expression in a lineage specific manner. In an earlier study [21], we have also observed that non-synonymous variations were also significantly overrepresented within the L2 gene of CaCx cases harboring intact HPV16 genomes, irrespective of lineage. Taken together, like E6, E7 and E5, the aberrant L2 gene could potentially play an oncogenic role in CaCx cases portraying episomal HPV16, as opposed to those harboring viral integration. Overall, this study also leads us to confirm that multiple pathways other than E2 disruption could be associated with CaCx pathogenesis. Perhaps, this is likely to involve characteristically different sets of host genes and pathways, than those recorded among cases with integrated viral genomes in the light of expression of L2 in the former case types as opposed to those in the latter. Finally, our study implicates that the L2 gene might serve as a novel biomarker or a target for those cases harboring episomal HPV16 genomes with intact E2 gene.

## Supporting Information

Figure S1 **Representative electropherograms showing the single nucleotide polymorphism, SNP (T4228C) within the non-coding region 2 (NCR2) of E2 intact/episomal (episomal or concomitant) HPV16 variants.** Region sequenced (nt 4216–4240) covers a part of NCR2 and L2 genes. The SNP (T4228C) is absent within the non malignant European variant sample and Asian American CaCx variant samples.
(TIF)

Figure S2 **Partition function analysis of the presence of T4228C SNP in the 5′UTR and its association with stability of the ensemble of mRNAs harboring NCR2 and L2 gene sequences in HPV16 positive (AA and E variant isolates) CaCx cases harboring episomal viral genomes.** The partition function matrix illustrates the base-pairing probabilities represented by dots. (a) Schematic representation of NCR2 (4139–4236) and a portion of the L2 gene with the T4228C SNP indicated in green; (b) Partition function heat map of the transcripts in absence of the SNP (T4228C); (c) Partition function heat map of the transcripts in presence of the SNP (T4228C); (d) Nucleotide base-pair probability (or accessibility) of the 5′ UTR (NCR2) of L2 mRNA without SNP (black) and with SNP (red).The position of the variation is marked in green. The Pearson correlation coefficient was 0.99 between (b) and (c).
(TIF)

Figure S3 **Representative electropherograms showing the SNP (G7193T) within the negative regulatory RNA element (LRE) of E2 intact/episomal (episomal or concomitant) HPV16 variants.** Region sequenced (7179–7207 bp) covers a part of LRE and LCR. The SNP (G7193T) is absent only within the non-malignant European variant samples.
(TIF)

Table S1 **Primer sequences and PCR conditions for re-sequencing of E1 ORF.**
(DOC)

Table S2 **Synonymous variations leading to humanized codons in E6, E5 and L2 ORFs.**
(DOC)

Table S3 **miRNA binding sites within the short non coding region (NCR2) (nt 4139–4236) of HPV16 genome.**
(DOC)

Table S4 **Impact of repeat variations within NCR2 (nt 4139–4236) on L2 RNA secondary structure.**
(DOC)
