# Supplementary figures and images for: Differential Expression of HPV16 L2 Gene in Cervical Cancers Harboring Episomal HPV16 Genomes: Influence of Synonymous and Non-Coding Region Variations
Source: PLoS One. 2013 Jun 6;8(6):e65647. doi: 10.1371/journal.pone.0065647 (PMC3675152; doi:10.1371/journal.pone.0065647)

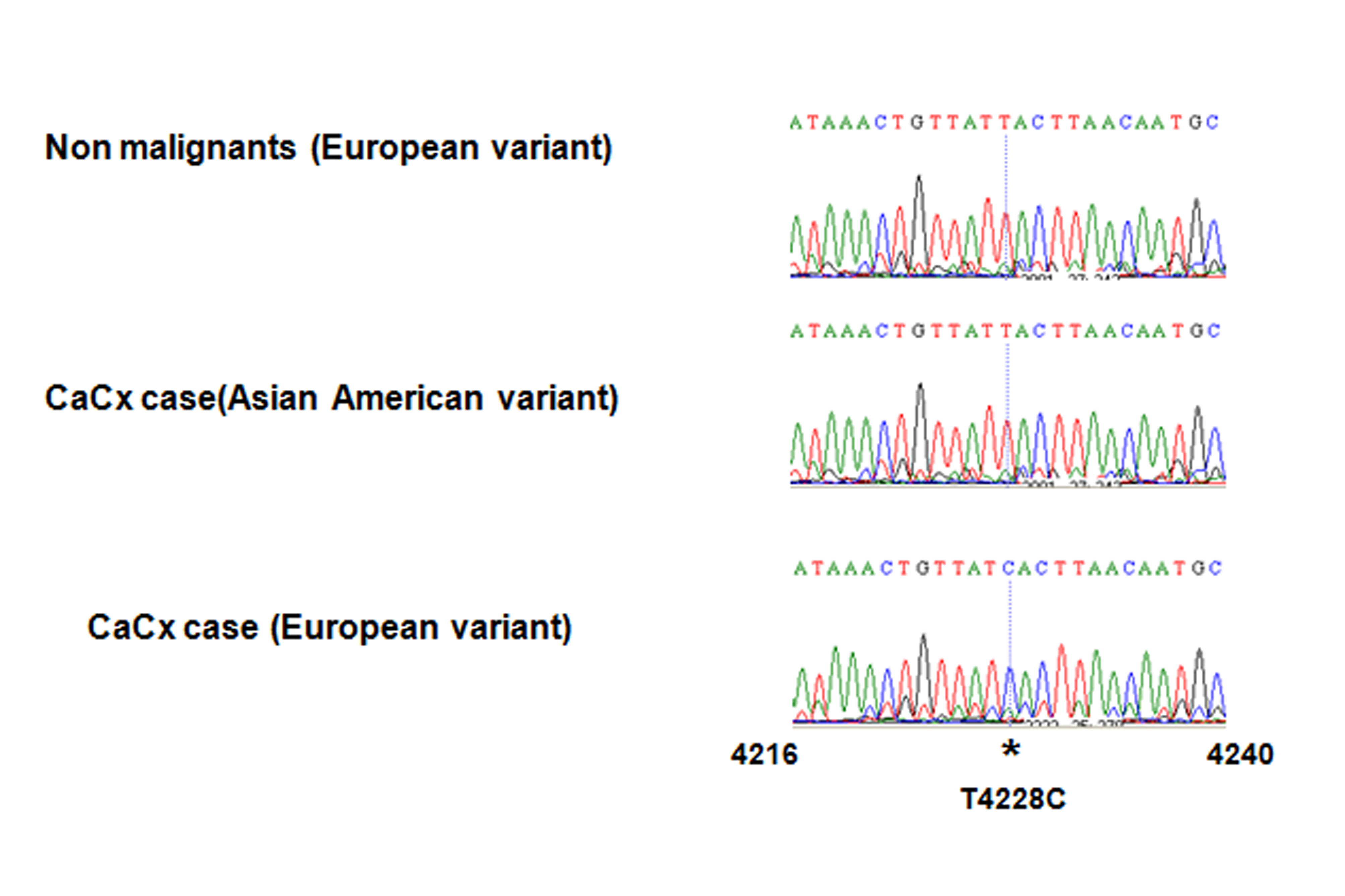

Supplement: Figure S1 — Representative electropherograms showing the single nucleotide polymorphism, SNP (T4228C) within the non-coding region 2 (NCR2) of E2 intact/episomal (episomal or concomitant) HPV16 variants. Region sequenced (nt 4216–4240) covers a part of NCR2 and L2 genes. The SNP (T4228C) is absent within the non malignant European variant sample and Asian American CaCx variant samples. (TIF) [file pone.0065647.s001.tif]

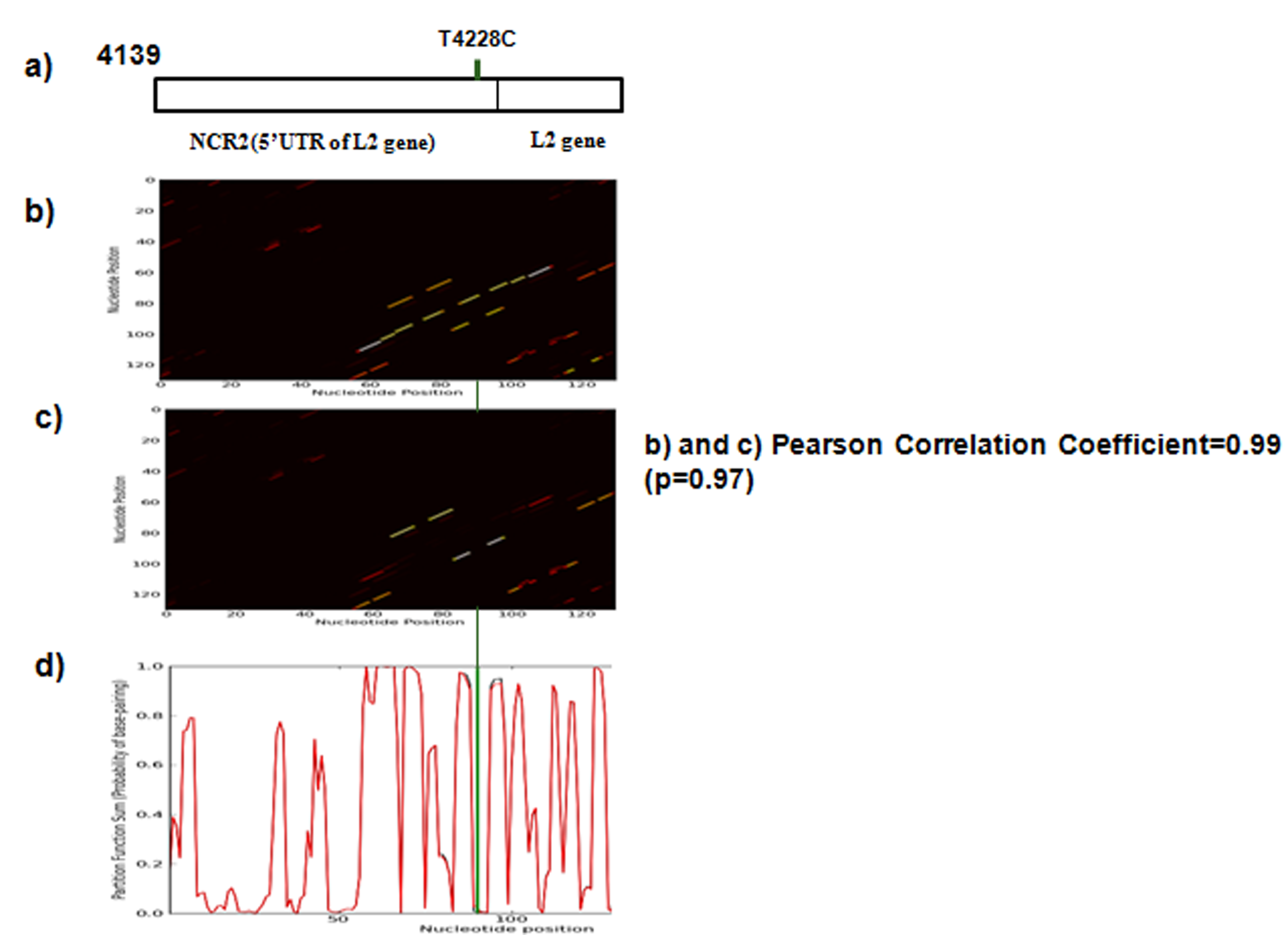

Supplement: Figure S2 — Partition function analysis of the presence of T4228C SNP in the 5′UTR and its association with stability of the ensemble of mRNAs harboring NCR2 and L2 gene sequences in HPV16 positive (AA and E variant isolates) CaCx cases harboring episomal viral genomes. The partition function matrix illustrates the base-pairing probabilities represented by dots. (a) Schematic representation of NCR2 (4139–4236) and a portion of the L2 gene with the T4228C SNP indicated in green; (b) Partition function heat map of the transcripts in absence of the SNP (T4228C); (c) Partition function heat map of the transcripts in presence of the SNP (T4228C); (d) Nucleotide base-pair probability (or accessibility) of the 5′ UTR (NCR2) of L2 mRNA without SNP (black) and with SNP (red).The position of the variation is marked in green. The Pearson correlation coefficient was 0.99 between (b) and (c). (TIF) [file pone.0065647.s002.tif]

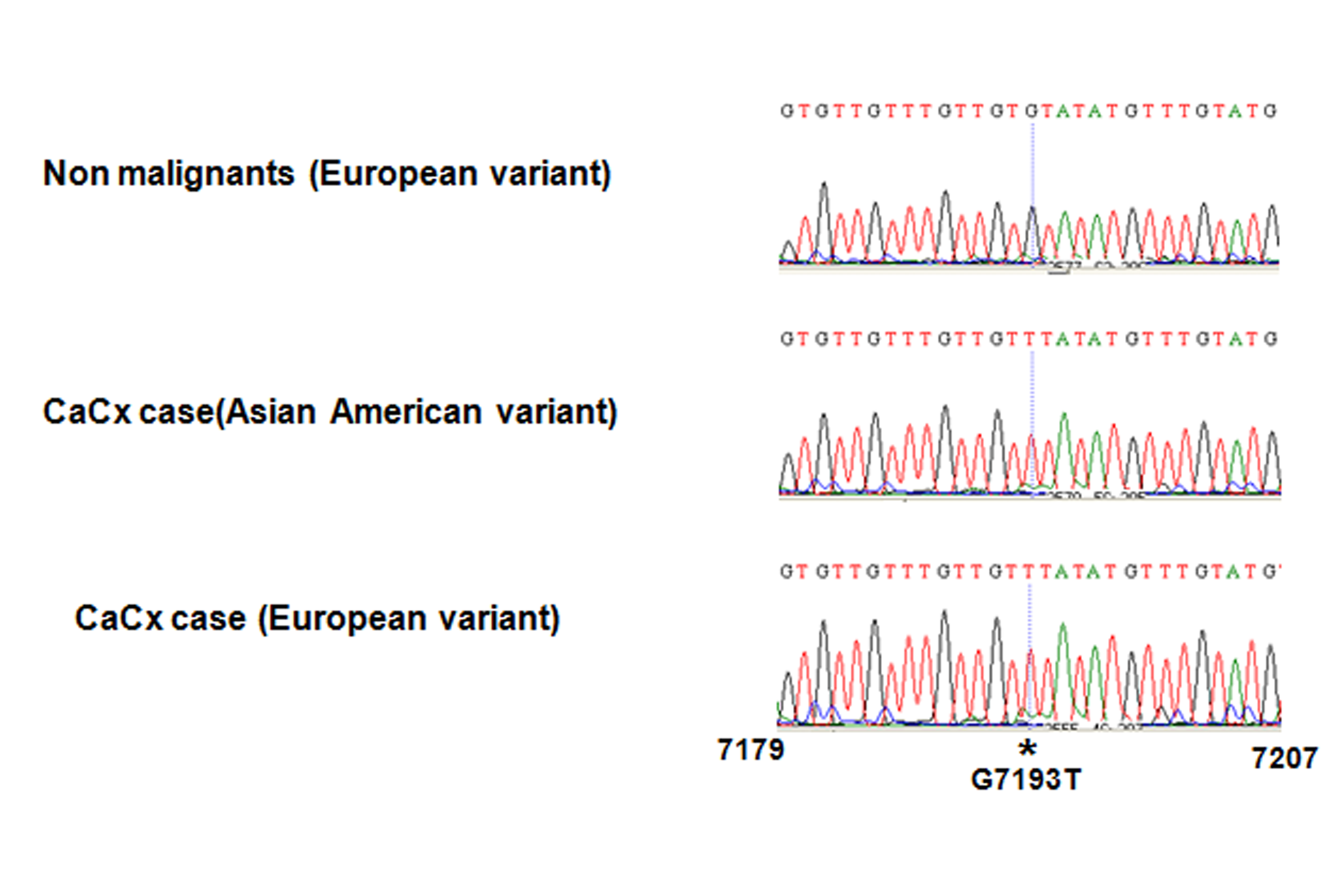

Supplement: Figure S3 — Representative electropherograms showing the SNP (G7193T) within the negative regulatory RNA element (LRE) of E2 intact/episomal (episomal or concomitant) HPV16 variants. Region sequenced (7179–7207 bp) covers a part of LRE and LCR. The SNP (G7193T) is absent only within the non-malignant European variant samples. (TIF) [file pone.0065647.s003.tif]
