# Supplementary material for: Differential Expression of HPV16 L2 Gene in Cervical Cancers Harboring Episomal HPV16 Genomes: Influence of Synonymous and Non-Coding Region Variations
Source: PLoS One. 2013 Jun 6;8(6):e65647. doi: 10.1371/journal.pone.0065647 (PMC3675152; doi:10.1371/journal.pone.0065647)
Supplement: Table S1 — Primer sequences and PCR conditions for re-sequencing of E1 ORF. (DOC) [file pone.0065647.s004.doc]

| **Re-sequencing primers(5'-3')** | **Position** | **Product (bp)** | **Conditions** |
| --- | --- | --- | --- |
| **E1(1)F:** TTA ACA CAG GCA GAA ACA GAG ACA  **E1(1)R:** CCA AAT GCA GCA ATA CAC CAA T | 1040  1590 | 551 | Denaturation: 95 °C/30 s  Annealing: 51 °C/30 s  Extension: 72 °C/45 s |
| **E1(2)F:** TAT TGC TGC ATT TGG ACT TAC AC  **E1(2)R:** CAC CTC CAT CAT CTA CCC TAT CA | 1576  2177 | 602 | Denaturation: 95 °C/30 s  Annealing: 48 °C/30 s  Extension: 72 °C/45 s |
| **E1(3)F:** TAG ATG TGA TAG GGT AGA TGA TGG  **E1(3)R:** ACT GGA TTT CCG TTT TCG TC | 2149  2667 | 519 | Denaturation: 95 °C/30 s  Annealing: 47 °C/30 s  Extension: 72 °C/45 s |
| **E1(4)F:** TGA AAT TTC TGC AAG GGT CTG TAA  **E1(4)R:** TAG GCG CAT GTG TTT CCA ATA G | 2337  2870 | 534 | Denaturation: 95 °C/30 s  Annealing: 51 °C/30 s  Extension: 72 °C/45 s |
