# Supplementary material for: Differential Expression of HPV16 L2 Gene in Cervical Cancers Harboring Episomal HPV16 Genomes: Influence of Synonymous and Non-Coding Region Variations
Source: PLoS One. 2013 Jun 6;8(6):e65647. doi: 10.1371/journal.pone.0065647 (PMC3675152; doi:10.1371/journal.pone.0065647)
Supplement: Table S2 — Synonymous variations leading to humanized codons in E6, E5 and L2 ORFs. (DOC) [file pone.0065647.s005.doc]

| **ORFs** | **Variations** | **MAF** | **Codon change** | **Codon usage change (%)** | **Amino acid change** | **Position of amino acid** | Domain |
| --- | --- | --- | --- | --- | --- | --- | --- |
| **E6** | A289G | 0.14 | GTA-GTG | 12-46 | V-V | 68 | - |
|  | A532G | 0.06 | TCA-TCG | 5-15 | S-S | 150 | - |
| **E5** | T3858C | 0.14 | AAT-AAC | 47-53 | N-N | 3 | - |
|  | T4089C | 0.14 | TTT-TTC | 46-54 | F-F | 80 | - |
| **L2** | A4242G | 0.01 | CGA-CGG | 11-20 | R-R | 2 | DBD |
|  | A4266C | 0.02 | ACA-ACC | 28-36 | T-T | 10 | Furin-CS+DBD |
|  | T4281C | 0.14 | GCT-GCC | 27-40 | A-A | 15 | CSBM |
|  | A4299C | 0.01 | ACA-ACC | 28-36 | T-T | 21 | CSBM |
|  | A4413C | 0.01 | GGA-GGC | 25-34 | G-G | 59 | - |
|  | T4446G | 0.01 | ACT-ACG | 11-25 | T-T | 70 | - |
|  | T4452C | 0.14 | TAT-TAC | 44-56 | Y-Y | 72 | - |
|  | A4467C | 0.02 | ACA-ACC | 28-36 | T-T | 77 | - |
|  | T4527C | 0.01 | CCT-CCC | 11-32 | P-P | 97 | - |
|  | T4545G | 0.01 | CCT-CCG | 11-29 | P-P | 103 | - |
|  | T4572G | 0.01 | ACT-ACG | 11-25 | T-T | 112 | CRE |
|  | A4599C | 0.07 | ACA-ACC | 28-36 | T-T | 121 | CRE |
|  | A4632C | 0.02 | GGA-GGC | 25-34 | G-G | 132 | L1-ID |
|  | T4647G | 0.011 | ACT-ACG | 11-25 | T-T | 137 | L1-ID |
|  | T4695C | 0.01 | ACT-ACC | 11-36 | T-T | 153 | L1-ID |
|  | A4788C | 0.02 | TCA-TCC | 15-22 | S-S | 184 | L1-ID |
|  | A4887G | 0.08 | CCA-CCG | 11-28 | P-P | 217 | L1-ID |
|  | A4944G | 0.03 | AAA-AAG | 43-57 | K-K | 236 | L1-ID |
|  | A4950G | 0.08 | GTA-GTG | 12-46 | V-V | 238 | L1-ID |
|  | A5187C | 0.01 | GGA-GGC | 25-34 | G-G | 317 | - |
|  | T5403C | 0.14 | TCT-TCC | 19-22 | S-S | 389 | L1-ID |
|  | T5412G | 0.03 | GGT-GGG | 16-25 | G-G | 392 | L1-ID |
|  | T5478C | 0.01 | CCT-CCC | 29-32 | P-P | 414 | L1-ID |
|  | T5523C | 0.05 | CCT-CCC | 29-32 | P-P | 429 | L1-ID |
|  | A5532G | 0.11 | CCA-CCG | 11-28 | P-P | 432 | L1-ID |

**DBD=DNA BINDING DOMAIN,Furin-CS= Furin CLEAVAGE SITE, CSBM= CELL SURFACE BINDING MOTIF, CRE= CROSS REACTIVE EPITOPE, L1-ID= L1 INTERACTION DOMAIN**
