# Supplementary material for: Differential Expression of HPV16 L2 Gene in Cervical Cancers Harboring Episomal HPV16 Genomes: Influence of Synonymous and Non-Coding Region Variations
Source: PLoS One. 2013 Jun 6;8(6):e65647. doi: 10.1371/journal.pone.0065647 (PMC3675152; doi:10.1371/journal.pone.0065647)
Supplement: Table S3 — miRNA binding sites within the short non coding region (NCR2) (nt 4139–4236) of HPV16 genome. (DOC) [file pone.0065647.s006.doc]

| **No.** | **miRNA ID** | **Location of binding sites within NCR2 of HPV16 genome** | **Length** | **Minimum Free Energy(kcal/mol)** | **Score for hybridization** |
| --- | --- | --- | --- | --- | --- |
| 1 | hsa-miR-3148 | 4146~4166 | 21 | -8.2 | 145 |
| 2 | hsa-miR-3174 | 4139~4157 | 19 | -13.2 | 146 |
| 3 | hsa-miR-3613-3p | 4174~4197 | 24 | -8.5 | 161 |
| 4 | hsa-miR-3916 | 4144~4169 | 26 | -14.2 | 147 |
| 5 | hsa-miR-495 | 4191~4211 | 21 | -7.6 | 148 |
| 6 | hsa-miR-548a-5p | 4214~4234 | 21 | -7.1 | 145 |
| 7 | hsa-miR-548b-5p | 4213~4234 | 22 | -7.1 | 145 |
| 8 | hsa-miR-548c-5p | 4212~4234 | 23 | -10.3 | 152 |
| 9 | hsa-miR-548d-5p | 4212~4234 | 23 | -7.1 | 148 |
| 10 | hsa-miR-548h-5p | 4212~4234 | 23 | -10.3 | 152 |
| 11 | hsa-miR-548i-5p | 4212~4234 | 23 | -8 | 144 |
| 12 | hsa-miR-548j | 4212~4234 | 23 | -8.6 | 144 |
| 13 | hsa-miR-548w | 4211~4234 | 24 | -9 | 144 |
| 14 | hsa-miR-548y | 4212~4234 | 23 | -7.7 | 152 |

**Presence of the SNP (T4228C) within NCR2 of E variants results in loss of 9 miRNA binding sites (hsa-miR-548a-5p, hsa-miR-548b-5p, hsa-miR-548c-5p, hsa-miR-548d-5p, hsa-miR-548h-5p, hsa-miR-548i-5p, hsa-miR-548j, hsa-miR-548w, hsa-miR-548y) on the basis of minimum free energy (<-7kcal/mol) and hybridization score(>140).**
