# Supplementary material for: Differential Expression of HPV16 L2 Gene in Cervical Cancers Harboring Episomal HPV16 Genomes: Influence of Synonymous and Non-Coding Region Variations
Source: PLoS One. 2013 Jun 6;8(6):e65647. doi: 10.1371/journal.pone.0065647 (PMC3675152; doi:10.1371/journal.pone.0065647)
Supplement: Table S4 — Impact of repeat variations within NCR2 (nt 4139–4236) on L2 RNA secondary structure. (DOC) [file pone.0065647.s007.doc]

| **HPV16 variants** | **Repeat variations in NCR2(4185-4213)** | **SNP(T4228C) in NCR2** | **Minimum free energy for optimal RNA secondary structure**  **(kcal/mol)** |
| --- | --- | --- | --- |
| **European variant type 1** | ATTTTTTTTTTT[GTTT]_3_  (GTTTTTTAATAAA) | Absent | -330.9 |
| **European variant type 2** | ATTTTTTTTTTT[GTTT]_3_  (GTTTTTTAATAAA) | Present | -331.1 |
| **European variant type 3** | ATTTTTTTTTTT[GTTT]_4_  (GTTTTTTAATAAA) | Absent | -332.1 |
| **European variant type 4** | ATTTTTTTTTTT[GTTT]_4_  (GTTTTTTAATAAA) | Present | -331.1 |
| **European variant type 5** | ATTTTTTTTTTT[GTTT]_5_  (GTTTTTTAATAAA) | Absent | -335 |
| **European variant type 6** | ATTTTTTTTTTT[GTTT]_5_  (GTTTTTTAATAAA) | Present | -334 |
| **Asian American variant type 1** | ATTTTTTTTTTT[ATTTTT]_3_  (GTTTTTTAATAAA) | Absent | -321.4 |
| **Asian American variant type 2** | ATTTTTTTTTTT[ATTTTT]_4_  (GTTTTTTAATAAA) | Absent | -321.4 |
| **Asian American variant type 3** | ATTTTTTTTTTT[ATTTTT]_5_  (GTTTTTTAATAAA) | Absent | -321.4 |
| **Asian American variant type 4** | GTTTTTTTTCTTATTTGTGT  (GTTTTTTAATAAA) | Absent | -329.1 |
